# De Novo *ACTG1* Variant Expands the Phenotype and Genotype of Partial Deafness and Baraitser–Winter Syndrome

**DOI:** 10.3390/ijms23020692

**Published:** 2022-01-08

**Authors:** Mateusz Dawidziuk, Anna Kutkowska-Kazmierczak, Ewelina Bukowska-Olech, Marta Jurek, Ewa Kalka, Dorothy Lys Guilbride, Mariusz Ireneusz Furmanek, Monika Bekiesinska-Figatowska, Jerzy Bal, Pawel Gawlinski

**Affiliations:** 1 Department of Medical Genetics, Institute of Mother and Child, 01-211 Warsaw, Poland; mateusz.dawidziuk@imid.med.pl (M.D.); marta.jurek@imid.med.pl (M.J.); jerzy.bal@imid.med.pl (J.B.); 2 Department of Medical Genetics, Poznan University of Medical Sciences, 60-806 Poznan, Poland; ewe.olech@gmail.com; 3 Unit of Anthropology, Institute of Mother and Child, 01-211 Warsaw, Poland; ewa.kalka@imid.med.pl; 4 Independent Researcher, Manhiça MPT 1122, Mozambique; lys.guilbride@gmail.com; 5 Department of Radiology and Diagnostic Imaging, Centre for Postgraduate Medical Education, 02-507 Warsaw, Poland; m.i.furmanek@interia.pl; 6 Department of Diagnostic Imaging, Institute of Mother and Child, 01-211 Warsaw, Poland; monika.bekiesinska@imid.med.pl

**Keywords:** *ACTG1* gene, Baraitser–Winter syndrome, partial deafness, actinopathy

## Abstract

Actin molecules are fundamental for embryonic structural and functional differentiation; γ-actin is specifically required for the maintenance and function of cytoskeletal structures in the ear, resulting in hearing. Baraitser–Winter Syndrome (B-WS, OMIM #243310, #614583) is a rare, multiple-anomaly genetic disorder caused by mutations in either cytoplasmically expressed actin gene, *ACTB* (β-actin) or *ACTG1* (γ-actin). The resulting actinopathies cause characteristic cerebrofrontofacial and developmental traits, including progressive sensorineural deafness. Both *ACTG1*-related non-syndromic A20/A26 deafness and B-WS diagnoses are characterized by hypervariable penetrance in phenotype. Here, we identify a 28th patient worldwide carrying a mutated γ-actin *ACTG1* allele, with mildly manifested cerebrofrontofacial B-WS traits, hypervariable penetrance of developmental traits and sensorineural hearing loss. This patient also displays brachycephaly and a complete absence of speech faculty, previously unreported for *ACTG1*-related B-WS or DFNA20/26 deafness, representing phenotypic expansion. The patient’s exome sequence analyses (ES) confirms a de novo *ACTG1* variant previously unlinked to the pathology. Additional microarray analysis uncover no further mutational basis for dual molecular diagnosis in our patient. We conclude that γ-actin c.542C > T, p.Ala181Val is a dominant pathogenic variant, associated with mildly manifested facial and cerebral traits typical of B-WS, hypervariable penetrance of developmental traits and sensorineural deafness. We further posit and present argument and evidence suggesting *ACTG1*-related non-syndromic DFNA20/A26 deafness is a manifestation of undiagnosed *ACTG1*-related B-WS.

## 1. Introduction

Actin isoforms β and γ are identical except for four amino acid substitutions within the first 10 N-terminal residues. Both isoforms are vital to embryonic differentiation and interact in some tissues, with each having hundreds of first-level interactions with other cellular molecules [1]. Nonetheless, these forms have different specific expression patterns and are involved in multiple, largely separate processes, with little functional redundancy [1,2]. γ-Actin expression predominates in the auditory hair cells [3] and is required for the formation and maintenance of the structures involved in the mechanotransduction of sound waves to electrical signals which are relayed to the brain as hearing [4,5,6,7]. Consistent with differential expression patterns, the accumulated data support phenotype–genotype correlation for B-WS-associated mutations in *ACTG1* versus *ACTB* (OMIM#102560 and #102630). Clinically, both *ACTB*-related and *ACTG1*-related B-WS share characteristic dysmorphic frontofacial traits: hypertelorism, ptosis, wide face and frequently, trigonocephaly. Both also present with a range of developmental traits, including sensorineural deafness, iris and retinal coloboma, growth retardation and intellectual disability. Cleft palate, hallux duplication and congenital heart problems are more common in *ACTB*-related BW-S. However, *ACTG1*-related B-WS patients show milder and highly variable trait manifestation [8,9,10] and varying degrees of deafness and time of onset, as well as other, predominantly neuromigration-related developmental traits. These include intellectual disability, microcephaly, coloboma of the iris and lens or/and optic nerve, optic nerve anomalies and structural/microstructural changes in the brain, such as pachygyria, myelination defects, lissencephaly and microlissencephaly [8]. Hypervariable penetrance associated with *ACTG1*-related disease means that any, all or possibly even none of these may be apparent in a patient carrying a pathogenic *ACTG1* allele. 

The correlation of different intragenic point mutations, or actin protein domains, with specific phenotypic features, however, remains unclear. 

The majority (28/51) of confirmed pathogenic *ACTG1* mutations in the Human Gene Mutation Database (HGMD) are reported to be associated with non-syndromal sensorineural hearing loss (NSHL) DFNA20/26 (Table 1).

In the literature to date, these variants do not overlap with the 17/51 confirmed B-WS-related *ACTG1* variants [53]. The two conditions are therefore considered to be separate molecular and clinical diagnoses. Pathology that is neither B-WS nor deafness has been reported for 13 other listed *ACTG1* variants (2, 12, 13, 14, 23, 27, 31, 37, 40, 46, 47, 49 and 69; Table 1). Pathogenic or possibly pathogenic variants listed for *ACTG1* therefore currently fall into three non-overlapping subsets according to the reported phenotype, with causal implications. However, the accumulated literature [10,24,50] reveals a snowballing degree of wobble in the molecular classification of *ACTG1* variants into subsets causing different pathologies. As individual *ACTG1*-related B-WS cases and familial DFNA20/26 studies accumulate, as well as *ACTG1*-linked pathologies reported as unassociated with either, the overlap of the symptoms and molecular etiology has become increasingly evident. A compilation of the data to date in Table 1 presents this information, which is taken up in our discussion. This has considerable diagnostic, clinical and genetic counseling impact, as well as biomedical interest. Here we present a 28th B-WS patient carrying a previously unassociated mutation in *ACTG1* that encapsulates and informs this issue. 

## 2. Results

Our patient is a boy born at term in the 39th week of the mother’s third pregnancy by natural delivery. Parents (father: 40 years, healthy; mother: 39 years at date of birth, treated with Plaquenil for lupus erythematosus diagnosed 3 years earlier) are non-consanguineous Caucasians of Polish descent. During pregnancy, nuchal translucency at the 95th percentile and a higher risk of trisomy 21 was determined by a non-invasive test (PAPP-A), and a cell-free fetal DNA (cffDNA) test was performed. This showed a low risk of Down syndrome.

### 2.1. Patient Parameters at Birth

Patient parameters at birth were as follows: weight, 3350 g (50 c); length, 56 cm (>95 c); occipital frontal circumference (OFC), 34 cm (25 c); Apgar score, 10; head circumference, 34 cm (−2 SD of the normal mean). Distinctive dysmorphic features and cryptorchidism were noted; an ultrasound examination revealed unilateral duplication of the pelvicalyceal system and echocardiography showed a membranous ventricular septal defect (VSD). Ultrasound images of all other internal organs were normal. The patient tested negative at birth for toxoplasmosis and cytomegaly.

**Figure 1:**
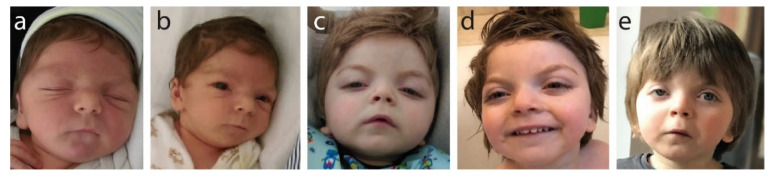
Photographic documentation of frontofaciocerebral features: (**a**) 3 months old (hypertelorism, short nose, round face); (**b**) 8 months old (small chin, retrognathia, posteriorly rotated ears); (**c**) 12 months old (arched eyebrows, elongated palpebral fissures, short nose, mouth corners directed down, round face); (**d**) 2 years and 2 months old (high forehead and frontal bossing); (**e**) 4 years old (unilateral ptosis, visible changing of the face’s shape with age, which is becoming less round with more visible arched eyebrows).

Facial features noted at birth and as the child grew (Figure 1) show wide-set eyes (hypertelorism), microcephaly and a wide face. 

Clinically, these specific facial features presenting together are strongly indicative of B-WS. Brachycephaly, a short nose and a long philtrum were also noted at birth and were present at all later examination age points. Progressively, we saw the following: at age 3 months: hypertelorism, short nose and round face; at age 8 months: small chin, retrognathia and posteriorly rotated ears; at age 12 months: progressive and more pronounced arching of the eyebrows, elongated palpebral fissures, mouth corners directed down, face becoming less round and more elongated, and unilateral ptosis; at age 2 years 2 months: high forehead with frontal bossing evident; at age 4years: strong unilateral ptosis, more pronounced arching of the eyebrows and face shape changing with age, becoming increasingly less round. 

### 2.2. Sensorineural Deafness

Screening tests after birth suggested deafness. Hearing tests between the ages of 2 to 4 months were inconclusive. At 4 months, severe deafness was diagnosed using the Auditory Brainstem Response (ABR) test, with 70 dB (left) and 80–90 dB (right) auditory deficits. At age 3 and a half months, CT imaging revealed shadowing of the entire pneumatic structures of both pyramids, suggesting inner ear inflammation. His middle ear canals were drained, anti-inflammatory treatment was applied and bilateral hearing aids were used from 6 months. Brainstem auditory evoked potentials (BAEP) at 5 months and at 16 months were positive. Hearing tests at 3 years 4 months showed sensorineural hearing loss of medium degree with a reaction to sound at 35 dB and 40–45 dB with and without hearing aids, respectively. This reflects an appreciable regression of deafness, out of character for B-WS or isolated *ACTG1*-related deafness, which are both normally progressive. We conclude that early infection of the ear canals and subsequent healing were responsible for the regressive nature of deafness in our patient, since hearing improved after drainage and anti-inflammatory treatment. We note that congenital malformations of the skull (microcephaly, brachycephaly) may contribute to an anomalous inner ear structure causing secretion and fluid accumulation, and a predisposition to ear canal infections exacerbating hearing loss. 

**Figure 2:**
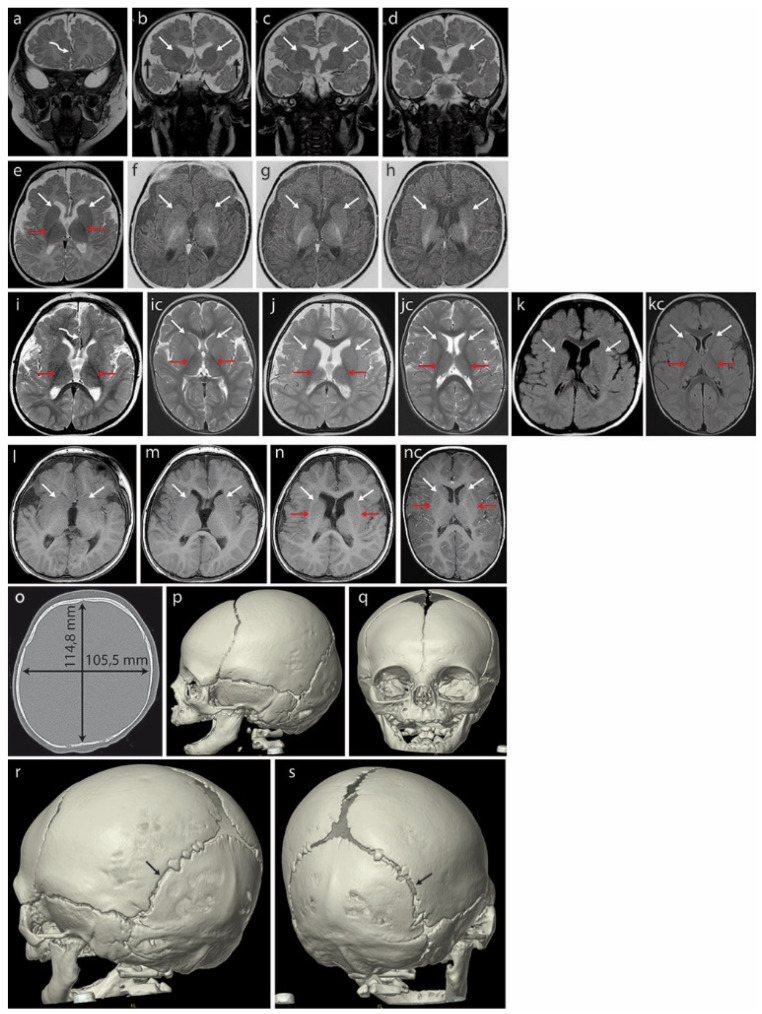
Brain MRI of the patient at 3 months (**a**–**h**) revealed hypoplasia/fenestration of the anterior falx cerebri with the gyri of the right cerebral hemisphere crossing the midline ((**a**)—curved arrow) and incomplete opercularization, which is normally complete at term ((**b**)—black arrows). Roundish basal ganglia in the coronal plane in T2-weighted images (T2WI) ((**b**–**d**)—white arrows). At this age, the posterior limbs of the internal capsules (PLICs—red arrows) are well myelinated: black in T2WI ((**e**)—axial plane) and white in the inversion recovery (IR) sequence, best depicting the myelinated structures (**f**–**h**). There is no trace of even the unmyelinated lines separating the caudate and lentiform nuclei that would represent the anterior limbs of the internal capsules (ALICs) ((**b**–**h**)—white arrows). A further brain MRI scan at 18 months (**i**–**n**) did not detect the ALICs which are normally appreciable at 10–11 months. The basal ganglia appear to be fused bilaterally in both hemispheres ((**j**–**n**) white arrows in the axial plane: (**j**)—T2WI, (**k**)—FLAIR, (**l**–**n**)—T1WI); opercularization remained incomplete (**i**). Normal PLICs ((**i**)—red arrows). (**ic**) Corresponding sections in the normal brain of an 18-month-old boy, T2WI. PLICs—red arrows; normal ALICs separating the caudate and lentiform nuclei—white arrows. (**jc**) Corresponding sections in the normal brain of an 18-month-old boy, T2WI. PLICs—red arrows; normal ALICs separating the caudate and lentiform nuclei—white arrows. (**kc**) Corresponding sections in the normal brain of an 18-month-old boy, FLAIR. PLICs—red arrows; normal ALICs separating the caudate and lentiform nuclei—white arrows. (**nc**) Corresponding sections in the normal brain of an 18-month-old boy, T1WI. PLICs—red arrows; normal ALICs separating the caudate and lentiform nuclei—white arrows. Computed tomography (CT) of the skull at age 4 months (**o–s**) showed a brachycephalic skull (the cephalic index of 91.9 exceeded the range of 76 to 81 compatible with a mesaticephalic skull in normal males). Asymmetric lambdoid suture: narrower on the left (**r**—black arrow) compared with the wider right one (**s**—black arrow).

Magnetic resonance imaging (MRI) of the brain at 3 months (Figure 2a–h) revealed hypoplasia/fenestration of the anterior falx cerebri with the gyri of the right cerebral hemisphere crossing the brain midline (Figure 2a—curved arrow), as well as incomplete opercularization (Figure 2b—black arrows). 

Unusually rounded basal ganglia are visible in the coronal plane in T2-weighted images (T2WI) (Figure 2b–d, white arrows). The posterior limbs of the internal capsules (PLICs, red arrows) are well myelinated: these structures appear black in T2WI on the axial plane (Figure 2e) and white in inversion recovery (IR) sequences highlighting the myelinated structures (Figure 2f–h). The anatomical line normally separating the caudate and lentiform nuclei from the anterior limbs of the internal capsules (ALICs) remains undetectable at the structural and myelination levels (Figure 2b–h, white arrows), indicating severely disrupted/delayed myelination. This is currently considered rare in B-WS patients [8].

A brain MRI at 18 months (Figure 2i–n) showed that opercularization remained incomplete (Figure 2i, red arrows). The anterior limbs of the internal capsules (ALICs) that are normally appreciable at 10–11 months were not detected. The basal ganglia appear to be fused bilaterally (Figure 2j–n, arrows showing fusion lines).

A CT of the skull at age 4 months (Figure 2o–q) showed a brachycephalic skull with a cephalic index of 91.9, indicating pathologically disturbed proportions of the skull. This exceeded the range of 76 to 81 compatible with a mesaticephalic skull in normal males. A SD greater than 3.5 between the length and width of the skull was noted at 33 months (−2.29 SD vs. 1.32 SD). We note that the initial physical examination suggested positional brachycephaly with a discrete asymmetry of the occiput (plagiocephaly), but a later CT examination ruled out positional skull deformation because there were clear differences in the width of the lambdoid suture: it was narrow on the left side and wide on the right (Figure 2r,s).

A chest X-ray at 2 months showed an abnormal shape of the ribs (third to eighth).

### 2.3. Molecular Analyses

We used array comparative genomic hybridization (aCGH) to compare the patient’s and reference human genomes, and assess the genomic content variation and gene copy number aberrations. Our patient shows a normal genomic copy number and genomic content. 

**Figure 3:**
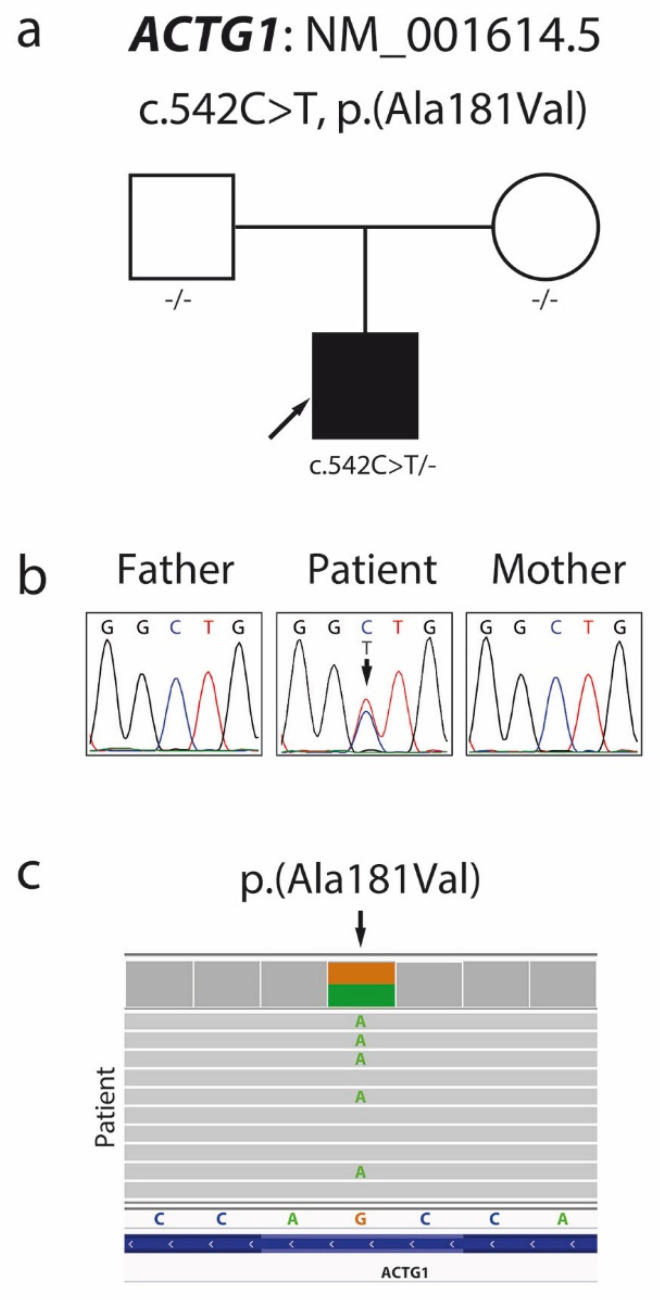
Pedigree (**a**) and Sanger sequencing chromatogram (**b**) showing segregation within the family and confirming the de novo character of the c.542C > T p.Ala181Val mutation in the *ACTG1* gene. Visual presentation of exome sequencing data made by the Integrative Genomics Viewer (**c**).

Patient exome screening for possible pathological mutations using exome sequencing (ES) identified a missense variant c.542C > T (NM_001614.5), p.Ala181Val (NP_001605.1) in the patient’s *ACTG1* gene (Figure 3c), confirmed by local Sanger sequencing. 

Family segregation analyses (Figure 3a,b) showed that the variant c.542C > T, p.Ala181Val arose de novo in one allele in our patient. In silico pathogenic prediction analysis resulted in 23 out of 25 prediction algorithms classifying this variant as deleterious, with high scores produced by the CADD and DANN algorithms (Table 2). The patient’s ES data revealed no additional pathogenic or likely pathogenic mutations to support dual molecular diagnoses for the patient’s phenotype.

## 3. Discussion

Our patient displays cranial, facial and developmental traits which together are clinically typical for B-WS [8]. These include microcephaly, specific dysmorphic facial features, intellectual disability, developmental defects of speech and brain microstructure, myelination defects and sensorineural hearing loss (Table 3, #14).

He also carries a de novo point mutation in the γ-actin gene *ATCG1* but no other mutation that could support a dual molecular diagnosis [14]. The specific point mutation identified here, c.542C > T, p.Ala181Val, was previously recorded in the dbSNP database (rs797044730) and the ClinVar clinical database (VCV000197198.4) as a variant with conflicting interpretations of pathogenicity, with no specific phenotype described; it is currently unlinked to any pathology. There is no further mention in the general literature, and it is not present in population genomic databases gnomAD v2 and v3. A pathogenic likelihood score of “potentially deleterious” was obtained for this variant when assessed by 23/25 predictive algorithms (Table 2). According to the American College of Medical Genetics and Genomics (ACMG) classification, this variant is classified as pathogenic (PS2, PM2, PM5, PP2, PP3). Furthermore, basal rate germline nucleotide point mutations per generation result in only one or two coding sequence mutations in a given exome [14], and our exome analyses detected only c.542C > T, p.Ala181Val, a coding missense point mutation in *ACTG1*. All B-WS-associated alleles for *ACTG1* reported to date are missense point mutations (Table 1), and are usually de novo, although an inherited *ACTG1* mutation linked to non-syndromic DFNA20/A26 deafness and B-WS in the same family has been reported [10]. Taken together, these data indicate that the variant identified here is responsible for the characteristic B-WS traits present, including deafness, and contributes to the phenotype expansion observed. Neither brachycephaly nor a complete absence of speech faculty have been previously described in *ACTG1*-related B-WS patients, nor for *ACTG1*-related isolated sensorineural deafness cases.

We therefore identify and reclassify the variant c.542C > T, p.Ala181Val as a dominant pathogenic allele of the *ACTG1* gene that is causative for BW-S and also sensorineural deafness.

Our patient is the third person documented as carrying this particular variant of *ACTG1* and the 28th patient worldwide documented with an array of symptoms resulting from the expression of defective γ-actin molecules providing a clear diagnosis of B-WS. 

Currently, the *ACTG1* variants listed in HGMD (69 including the variant presented here) fall into three non-overlapping groups associated with different symptomology: sensorineural deafness DFNA20/26, B-WS and symptomology apparently unrelated to either deafness or B-WS (Table 1, white background, grey background and green background, respectively). This leads to distinct molecular and clinical diagnoses, prognoses, therapies and genetic counseling for each group.

However, we note that, for all 69 (confirmed pathogenic and possibly pathogenic) *ACTG1* variants recorded in the HGMD to date, the associated pathologies are fully consistent with the hypervariable penetrance of *ACTG1*-related B-WS traits. These include the apparently non-syndromic deafness-associated variants (37/69), variants carrying a confirmed B-WS diagnosis (19/69) and variants reporting pathologies other than deafness or B-WS (13/69; variants 2, 12, 13, 14, 23, 27, 31, 37, 40, 46, 47, 49 and 69 in Table 1). Without exception, the specific disparate phenotypes reported for these (microcephaly, polymicrogyria, pachygyria, microlissencephaly, intellectual disability, coloboma of the eye, congenital diaphragmatic hernia, autism, multiple congenital anomalies) are consistent with hypervariable penetrance of the phenotypic spectrum of B-WS. 

There are also nine positions in the amino acid sequence of *ACTG1* (Table 1, inverted arrows; variants # 15/16, 18/19, 24/25, 33/34, 38/39, 41/42, 49/50, 56/57, 62/63) where two distinct substitutions arise from separate point mutations within the same codon. This leads to two different variants with an associated pathology listed in the HGMD. In most cases (7/9), the associated pathology is the same for both variants (deafness in 5/9 positions; B-WS in 2/9 positions); in two further cases (Table 1, positions p.181 and p.256), we see B-WS for one variant (#34, p.Ala181Val; #50 p.Arg256Trp), with either deafness (#33) or pachygyria (#49), respectively, for the alternative variant. Variant #34, (p.Ala181Val) represents our patient; different substitutions at the same position in *ACTG1* result in B-WS in our patient rather than isolated deafness, an apparently different phenotype. Deafness and pachygyria, however, are present in 76% and 89% respectively, of formally diagnosed *ACTG1*-related B-WS cases where an appropriate examination was performed (Table 1). We note that while a highly specific intragenic correlation of *ACTG1* mutation with a particular trait cannot at present be formally excluded without rigorous molecular mapping, the phenotype is identical for both variants in 7/9 of positions dispersed between positions p.82 and p.332 (77%) of the *ACTG1* protein sequence (375 residues). Further, in 9/9 positions, the phenotype is entirely consistent with hypervariable penetrance of traits within the spectrum for B-WS. 

In addition, familial studies for DFNA20/26 NSHL involving a single segregating variant (p.Ala58Val) for *ACTG1*-associated sensorineural deafness reflect both full-blown B-WS (with early onset, progressive deafness, ptosis, coloboma, seizures, developmental delay), a manifestation of isolated classic B-WS traits (seizures, early-onset progressive deafness) and apparently isolated non-syndromic deafness with anomalous optic nerves in successive generations of a family [10] (Table 2: #3–5).

In short, all mutations in *ACTG1* listed in the HGMD are associated with phenotypes consistent with hypervariable expression of B-WS traits. An extensive cellular network of first-level interaction partners exists for γ-actin molecules [1]. Together with normal variation in the patient’s genetic landscape, this implies considerable variability inherent in the mutation load impacting actin physiology and, therefore, actinopathies arising from a single pathogenic variant in different patient backgrounds. A simple interpretation is, therefore, that all these differing phenotypes, including deafness, reflect variable trait manifestation of undiagnosed B-WS. We therefore contend the phenotypes reported in 37/69 deafness-associated *ACTG1* variants, as well as the 18/69 confirmed variants with a pathology consistent with isolated B-WS traits (Table 1), though currently associated with neither B-WS nor deafness, result from variable penetrance of molecular variants underlying actinopathies capable of causing B-WS. 

Given the progressive nature and hypervariable trait penetrance of *ACTG1*-related B-WS and DFNA20/26 deafness, which are particularly well showcased in the familial context of segregating *ACTG1* pathogenic alleles, we hope that this perspective of the overlapping etiology may be clinically useful and, crucially, inform specific molecular diagnoses and genetic counseling, in addition to therapeutic handling.

## 4. Materials and Methods

**Array Comparative Genomic Hybridization (aCGH)**

We used the CGX 3x720K Whole-Genome array (Roche NimbleGen, Madison, WI, USA) as per the manufacturer’s instructions and scanned slides into image files using an MS 200 Microarray Scanner (Roche NimbleGen, Madison, WI, USA). Data were analyzed using DEVA (Roche NimbleGen, Madison, WI, USA) and Genoglyphix Analysis Software (Perkin Elmer, Waltham, MA, USA).

**Exome sequencing (ES)**

Genomic DNA was extracted from peripheral blood leukocytes of the patient and his parents using an automatic magnetic bead-based method using MagNA Pure 96 system, Roche, Germany. Whole-exome sequencing libraries were prepared using Agilent SureSelect XT Human All Exon V6 sample preparation kits and the Illumina NovaSeq 6000 sequencer (Illumina, CA, USA), via 2 × 100 bp reads. Genomic data processing was based on an in-house pipeline, and the reads were aligned with Burrows–Wheeler Aligner (BWA) v0.7.16 software to the GRCh38 reference genome. The Genome Analysis Toolkit (GATK) HaplotypeCaller v4.0b4 was used for variant calling and the Ensembl Variant Effect Predictor (VEP) v96 was used to annotate the variants. 

For variant prioritization, the ES data were first analyzed for the known pathogenic or likely pathogenic variants reported in the ClinVar database. The in silico gene panel was used for rare variants (under 0.01% in the gnomAD v2 database) in genes associated with microcephaly consisting of over 800 genes. Those genes were manually selected from several gene panels customized for patients with microcephaly, and additional genes were selected from various databases such as OMIM or DECIPHER. The detailed gene list is included in Supplementary Table S1. Variant pathogenicity prediction and ACMG classification were carried out with the use of the VarSome website [55] and the dnNSFP v4.2 database [56].

Polymerase chain reaction was performed by use of the FastStart Taq DNA Polymerase, dNTPack kit (Roche) as per the manufacturer’s instructions and the following primers: forward: TCCAGGTTTCTCATTTGGTTTCT; reverse: CCCGACAGCACCGTGTT (100 ng template DNA; annealing temperature, 58 °C; polymerase activity time, 1 min; 35 cycles; product length, 759 nt).

## Figures and Tables

**Table 1:** All *ACTG1* mutations listed in HGMD as of 30 November 2021. DM + red, disease-causing mutation; DM? + orange, possibly disease-causing mutation; B-WS + grey, Baraitser–Winter cerebrofrontofacial syndrome; green, mutations correlated with neither hearing loss nor B-WS. Nucleotide changes in the *ACTG1* gene are reported according to RefSeq transcript NM_001614.5. Amino acid changes are reported according to RefSeq transcript NP_001605.1. (↓) and (↑) indicate two different nucleotide substitutions within the same codon resulting in two variants.

No.	HGMD Number	Nucleotide Change	Amino Acid Change	Variant Class	Reported Phenotype
1	CM1611295	c.34A > G	p.Asn12Asp	DM	B-WS [9]
2	CM2015269	c.88G > T	p.Val30Leu	DM	Pachygyria [11]
3	CM181678	c.94C > T	p.Pro32Ser	DM	Hearing loss, non-syndromic [12]
4	CM208651	c.102C > G	p.Ile34Met	DM?	Hearing loss [13]
5	CM208650	c.110G > A	p.Arg37His	DM?	Hearing loss [13]
6	CM171610	c.118C > T	p.His40Tyr	DM?	B-WS [14]
7	CM153972	c.142G > C	p.Gly48Arg	DM	Deafness, dominant progressive [15]
8	CM094470	c.151G > A	p.Asp51Asn	DM	Deafness, dominant progressive [16]
9	CM175231	c.173C > T	p.Ala58Val	DM	B-WS [10]
10	CM208234	c.176A > G	p.Gln59Arg	DM	B-WS [17]
11	CM1821877	c.197C > T	p.Thr66Ile	DM	Hearing loss [18]
12	CM1710251	c.209C > T	p.Pro70Leu	DM	Ocular coloboma [19]
13	CM1827026	c.221G > T	p.Gly74Val	DM	Intellectual disability [20]
14	CM157593	c.223A > C	p.Ile75Leu	DM?	Microlissencephaly [21]
15	CM208652	c.246G > A	p.Met82Ile (↓)	DM?	Hearing loss [13]
16	CM164938	c.244A > T	p.Met82Leu (↑)	DM	Hearing loss [22]
17	CM032825	c.266C > T	p.Thr89Ile	DM	Deafness, dominant progressive [23]
18	CM094417	c.354G > C	p.Lys118Asn (↓)	DM	Deafness, dominant progressive [24]
19	CM032826	c.353A > T	p.Lys118Met (↑)	DM	Deafness, dominant progressive [23]
20	CM122513	c.359C > T	p.Thr120Ile	DM	B-WS [25]
21	CM085221	c.364A > G	p.Ile122Val	DM	Deafness, dominant progressive [26]
22	CM122514	c.404C > T	p.Ala135Val	DM	B-WS [25]
23	CM1931694	c.429C > T	p.Tyr143Tyr	DM?	Autism [27]
24	CM189438	c.434C > G	p.Ser145Cys (↓)	DM?	Sensorineural deafness, non-syndromic [28]
25	CM1724902	c.434C > T	p.Ser145Phe (↑)	DM	Hearing loss [29]
26	CM214253	c.439C > T	p.Arg147Cys	DM	B-WS [30]
27	CM157618	c.459G > C	p.Met153Ile	DM?	Microlissencephaly [21]
28	CM122515	c.464C > T	p.Ser155Phe	DM	B-WS [25]
29	CM1310523	c.485C > T	p.Thr162Met	DM	Deafness [31]
30	CM208653	c.493A > G	p.Ile165Val	DM?	Hearing loss [13]
31	CM175615	c.499G > A	p.Glu167Lys	DM?	Diaphragmatic hernia, congenital [32]
32	CM1611293	c.535G > T	p.Asp179Tyr	DM	B-WS [9]
33	CM164939	c.542C > G	p.Ala181Gly (↓)	DM	Hearing loss [22]
34	This report	c.542C > T	p.Ala181Val (↑)	DM	B-WS
35	CM189439	c.548G > A	p.Arg183Gln	DM?	Sensorineural deafness, non-syndromic [28]
36	CM127916	c.559G > C	p.Asp187His	DM	Hearing loss [33]
37	CM1618747	c.574A > T	p.Ile192Phe	DM	Multiple congenital anomalies [34]
38	CM122516	c.608C > A	p.Thr203Lys (↓)	DM	B-WS [25]
39	CM199153	c.608C > T	p.Thr203Met (↑)	DM	B-WS [17,35]
40	CM215619	c.616C > T	p.Arg206Trp	DM	Polymicrogyria [36]
41	CM1823137	c.628C > T	p.Arg210Cys (↓)	DM	B-WS [37]
42	CM1915915	c.628C > G	p.Arg210Gly (↑)	DM	B-WS [38]
43	CM161501	c.638A > G	p.Lys213Arg	DM	Hearing impairment, non-syndromic [39]
44	CM1918057	c.640G > A	p.Glu214Lys	DM	B-WS [40]
45	CM094418	c.721G > A	p.Glu241Lys	DM	Deafness, dominant progressive [24]
46	CM157614	c.728C > T	p.Pro243Leu	DM?	Microlissencephaly [21]
47	CM1722894	c.757G > A	p.Glu253Lys	DM?	Congenital heart disease [41]
48	CM122517	c.760C > T	p.Arg254Trp	DM	B-WS [25]
49	CM2015268	c.767G > A	p.Arg256Gln (↓)	DM	Pachygyria [11]
50	CM122518	c.766C > T	p.Arg256Trp (↑)	DM	B-WS [25]
51	CM171720	c.773C > T	p.Pro258Leu	DM	Hearing impairment [42]
52	CM032827	c.791C > T	p.Pro264Leu	DM	Deafness, dominant progressive [23]
53	CM1311076	c.802G > A	p.Gly268Ser	DM?	Hearing loss, early childhood [43]
54	CM208654	c.823C > T	p.His275Tyr	DM?	Hearing loss [13]
55	CM033588	c.833C > T	p.Thr278Ile	DM	Deafness, dominant progressive [44]
56	CM189440	c.848T > C	p.Met283Thr (↓)	DM?	Sensorineural deafness, non-syndromic [28]
57	CM1821805	c.847A > G	p.Met283Val (↑)	DM	Hearing loss [18]
58	CM1310318	c.895C > G	p.Leu299Val	DM	Deafness [45]
59	CM132288	c.914T > C	p.Met305Thr	DM	Hearing loss, non-syndromic [46]
60	CM1412647	c.946G > A	p.Glu316Lys	DM	Hearing lose [47]
61	CM1412838	c.974T > A	p.Met325Lys	DM	Hearing loss [48]
62	CM032828	c.994C > G	p.Pro332Ala (↓)	DM	Deafness, dominant progressive [23]
63	CM163638	c.994C > T	p.Pro332Ser (↑)	DM	Hearing loss, sensorineural [49]
64	CM1611294	c.1000G > C	p.Glu334Gln	DM	B-WS [9]
65	CM1611296	c.1004G > A	p.Arg335His	DM	B-WS [9]
66	CM164940	c.1045C > A	p.Leu349Met	DM	Hearing loss [22]
67	CM063834	c.1109T > C	p.Val370Ala	DM	Deafness, dominant progressive [50]
68	CD168453	c.626_632delTGCGCGA	p.Val209Alafs*73	DM	Hearing loss, non-syndromic [51]
69	CN1927117	Duplication of 859 kb including the entire gene + 37 others		DM?	Autism spectrum disorder [52]

**Table 2:** Results of in silico variant analysis using various prediction algorithms.

Algorithm	Raw Score	Prediction
SIFT4G	0.001	Damaging
Polyphen2 HDIV	0.347	Benign
Polyphen2 HVAR	0.179	Benign
LRT	0.000	Deleterious
MutationTaster	1.000	Disease causing
MutationAssessor	4.780	High
FATHMM	−4.870	Damaging
PROVEAN	−3.140	Damaging
MetaSVM	1.132	Damaging
MetaLR	0.961	Damaging
MetaRNN	0.964	Damaging
M-CAP	0.965	Damaging
REVEL	0.954	Pathogenic
MutPred	0.822	Pathogenic
MVP	0.955	Pathogenic
PrimateAI	0.841	Damaging
DEOGEN2	0.978	Damaging
BayesDel addAF	0.568	Damaging
BayesDel noAF	0.578	Damaging
ClinPred	0.998	Damaging
LIST S2	0.968	Damaging
FATHMM MKL	0.969	Damaging
FATHMM XF	0.961	Damaging
EIGEN	0.610	Pathogenic
EIGEN PC	0.600	Pathogenic
CADD	26.7	-
DANN	0.981	-

**Table 3:** List of all 28 published patients with deleterious mutations in the *ACTG1* gene and B-WS syndrome. Nd—no data; na—not applicable; R—regressive; CC–corpus callosum; F—female; M—male; F*—female proband in the study; F∆—mother of F*; M□—father of F*; M1 and M2—first and second patients from one study, red and bold—patient first described in this study.

#	Nucleotide Change	Amino Acid Change	Inheritance	Sex	Population	Short Stature	ID	Hearing Loss	Absence of Speech	Seizures	Micro-Cephaly	Trigonocephaly	Brachycephaly	Hypertelorism	High-arched Eyebrows	Ptosis	Iris or Retina Coloboma	Central Nervous System
1	c.34A > G	p.Asn12Asp	de novo	F	nd	+	+	−	−	−	−	nd	nd	nd	nd	+	−	No MRI [9]
2	c.118C > T	p.His40Tyr	de novo	M	nd	nd	nd	nd	nd	nd	nd	nd	nd	nd	nd	nd	nd	nd [14]
3	c.173C > T	p.Ala58Val	parental	F*	nd	nd	nd	+	nd	nd	nd	nd	nd	nd	nd	−	−	nd [10]
4	c.173C > T	p.Ala58Val	nd	F∆	nd	nd	nd	+	nd	nd	nd	nd	nd	nd	nd	−	−	nd [10]
5	c.173C > T	p.Ala58Val	nd	M□	nd	nd	nd	+	nd	nd	nd	nd	nd	nd	nd	+	+ (iris and retina)	nd [10]
6	c.176A > G	p.Gln59Arg	de novo	M1	Mexican	+	+	nd	−	nd	−	nd	nd	+	+	+	+ (iris, retina, optic nerve head)	Generalized decrease in the cerebral sulci and gyri compatible with pachygyria [17]
7	c.359C > T	p.Thr120Ile	de novo	F	nd	−	+	+	nd	+	−	+	nd	+	+	+	−	Pachygyria [25]
8	c.404C > T	p.Ala135Val	de novo	F	nd	+	+	+	nd	+	+	+	nd	−	+	+	+	Pachygyria [25]
9	c.439C > T	p.Arg147Cys	de novo	nd	nd	nd	nd	nd	nd	nd	nd	nd	nd	nd	nd	nd	nd	nd [30]
10	c.464C > T	p.Ser155Phe	de novo	M	nd	−	nd	nd	nd	+	−	+	nd	+	nd	+	nd	Pachygyria [25]
11	c.464C > T	p.Ser155Phe	de novo	F	nd	+	+	−	nd	+	+	+	nd	+	+	+	+	Pachygyria [25]
12	c.464C > T	p.Ser155Phe	nd	F	nd	nd	nd	nd	nd	+	nd	nd	nd	+	+	+	+	Pachygyria [25]
13	c.535G > T	Asp179Tyr	nd	F	nd	+	+	+	−	−	nd	nd	nd	nd	nd	−	−	Anterior-predominant pachygyria, posterior band heterotopias, enlarged ventricles, prominent perivascular spaces [9]
**14**	**c.542C > T**	**p.Ala181Val**	**de novo**	**M**	**Polish**	**+**	**+**	**+ R**	**+**	**−**	**+**	**−**	**+**	**+**	**+**	**+**	**−**	**Delayed myelination in frontal brain; gyri of the right cerebral hemisphere the cross brain midline**
15	c.608C > A	p.Thr203Lys	de novo	M	nd	−	nd	+	nd	+	−	+	nd	+	+	+	−	Pachygyria [25]
16	c.608C > T	p.Thr203Met	de novo	F	nd	nd	na	na	na	na	+	nd	nd	nd	nd	na	nd	Post-mortem fetal investigation: agenesis of the CC, colpocephaly, bilateral posterior dilatation of the lateral ventricles, incomplete operculization of the sylvian fissures [35]
17	c.608C > T	p.Thr203Met	de novo	M2	Mexican	+	+	nd	nd	nd	+	nd	nd	+	+	+	+ (iris)	Cortical dysplasia with several areas of pachygyria, short and thick CC with rostral agenesis and hypoplastic cerebellar vermis [17]
18	c.628C > T	p.Arg210Cys	de novo	F	Korean	nd	−	nd	nd	nd	+	nd	nd	nd	nd	nd	nd	nd [54]
19	c.628C > T	p.Arg210Cys	de novo	nd	nd	nd	+	+	nd	nd	+	nd	nd	nd	nd	nd	nd	Brain anomalies (not specified) [37]
20	c.628C > G	p.Arg210Gly	de novo	F	Japanese	−	+	nd	nd	−	−	nd	nd	nd	nd	nd	nd	nd [38]
21	c.640G > A	p.Glu214Lys	de novo	M	nd	+	nd	nd	nd	nd	nd	nd	nd	nd	nd	nd	nd	nd [40]
22	c.760C > T	p.Arg254Trp	nd	M	nd	+	+	nd	−	−	nd	nd	nd	nd	nd	−	−	Anterior-predominant pachygyria, prominent perivascular spaces [9]
23	c.760C > T	p.Arg254Trp	de novo	M	nd	−	+	+	nd	−	+	+	nd	+	+	+	+	Pachygyria [25]
24	c.766C > T	p.Arg256Trp	nd	M	nd	+	+	−	−	−	+	nd	nd	nd	nd	−	−	Anterior-predominant pachygyria, posterior band heterotopias, agenesis of the CC, enlarged ventricles, prominent perivascular spaces [9]
25	c.766C > T	p.Arg256Trp	nd	M	nd	+	+	−	−	−	nd	nd	nd	nd	nd	+	−	Anterior-predominant pachygyria, posterior band heterotopias, mega-CC, enlarged ventricles, prominent perivascular spaces [9]
26	c.766C > T	p.Arg256Trp	de novo	M	nd	+	+	+	nd	+	+	+	nd	+	+	+	+	Pachygyria [25]
27	c.1000G > C	p.Glu334Gln	nd	M	nd	−	+	+ (20–40 dB)	−	−	−	nd	nd	nd	nd	+	−	Frontal dysgyria, enlarged ventricles, prominent perivascular spaces (mild) [9]
28	c.1004G > A	p.Arg335His	de novo	F	nd	−	+	+	−	−	−	nd	nd	nd	nd	−	−	Frontal dysgyria, enlarged ventricles [9]

## Data Availability

Data are contained within the article.

## Supplementary Materials

The following are available online at https://www.mdpi.com/article/10.3390/ijms23020692/s1.
